# Comparative effectiveness research on patients with acute ischemic stroke using Markov decision processes

**DOI:** 10.1186/1471-2288-12-23

**Published:** 2012-03-09

**Authors:** Darong Wu, Yefeng Cai, Jianxiong Cai, Qiuli Liu, Yuanqi Zhao, Jingheng Cai, Min Zhao, Yonghui Huang, Liuer Ye, Yubo Lu, Xianping Guo

**Affiliations:** 1 The 2nd Clinical Medical College of Guangzhou University of Chinese Medicine, the 2nd Affiliated Hospital of Guangzhou University of Chinese Medicine, Guangzhou, China; 2 Guangdong Provincial Academy of Chinese Medical Sciences, Guangzhou, China; 3 South China Normal University, Guangzhou, China; 4 Sun Yat-Sen University, Guangzhou, China; 5 School of Mathematics and Computational Science, Sun Yat-Sen University, 135, Xingangxi Road, Haizhu District, Guangzhou, Guangdong, China

**Keywords:** Markov decision processes, Acute ischemic stoke, Comparative effectiveness research, Traditional Chinese Medicine/integrative medicine

## Abstract

**Background:**

Several methodological issues with non-randomized comparative clinical studies have been raised, one of which is whether the methods used can adequately identify uncertainties that evolve dynamically with time in real-world systems. The objective of this study is to compare the effectiveness of different combinations of Traditional Chinese Medicine (TCM) treatments and combinations of TCM and Western medicine interventions in patients with acute ischemic stroke (AIS) by using Markov decision process (MDP) theory. MDP theory appears to be a promising new method for use in comparative effectiveness research.

**Methods:**

The electronic health records (EHR) of patients with AIS hospitalized at the 2^nd ^Affiliated Hospital of Guangzhou University of Chinese Medicine between May 2005 and July 2008 were collected. Each record was portioned into two "*state*-*action*-*reward*" stages divided by three time points: the first, third, and last day of hospital stay. We used the well-developed optimality technique in MDP theory with the finite horizon criterion to make the dynamic comparison of different treatment combinations.

**Results:**

A total of 1504 records with a primary diagnosis of AIS were identified. Only *states *with more than 10 (including 10) patients' information were included, which gave 960 records to be enrolled in the MDP model. Optimal combinations were obtained for 30 types of patient condition.

**Conclusion:**

MDP theory makes it possible to dynamically compare the effectiveness of different combinations of treatments. However, the optimal interventions obtained by the MDP theory here require further validation in clinical practice. Further exploratory studies with MDP theory in other areas in which complex interventions are common would be worthwhile.

## Background

Comparative effectiveness research (CER) is a way of identifying what works for which patients under which circumstances [1]. CER is not a single entity, it can take many forms, including cohort studies, literature systematic reviews, observational studies, and randomized controlled trials (RCTs) [1,2]. Non-randomized comparative clinical studies also play an important role in assessing the safety and effectiveness of medical interventions for routine practice. Recent attention to non-randomized comparative clinical studies in CER has focused on methodological issues [3,4]. Experts realize that there are methodological challenges for non-randomized comparative clinical studies that cannot be ignored, especially with the increased requirements for data analysis driven by the demand for real-world evidence. These challenges include [4] dealing adequately with multiple therapies and possible outcomes; an extremely heterogeneous baseline in terms of patient characteristics and setting; and confounding in studies that use different kinds of health databases. Methodology researchers have made great progress in the development and application of statistical methods for the description and analysis of CER data [5-7]. Such methods include using propensity score analysis to adjust for group differences [8,9], structural equation models and decomposition methods to identify how outcomes vary differentially with respect to patient characteristics and other factors for alternative treatment cohorts [10], and instrumental variable methods to address the problem of uncontrolled confounding [7,11-14]. However, the uncertainties in real-world systems that evolve dynamically with time have yet to be adequately identified.

Treatment with syndrome differentiation is considered the kernel of Traditional Chinese Medicine (TCM)[15], which means that therapeutic interventions are changed dynamically according to the variation of the state of the syndrome or disease over time. There is a general impression among Chinese medicine practitioners that treatments that change dynamically with syndrome differentiation and time are superior to those that remain unchanged. However, when TCM treatments are tailored to the individual patient, as is common practice, it is more difficult to assess their effectiveness than when they are applied to all patients in a standard manner in clinical studies. Methods that allow the researcher to model the uncertainties in real-world practice, and especially those that may dynamically change with time, are needed to describe TCM treatments and compare their effectiveness.

MDP theory is a versatile and powerful tool used to analyze sequential decision problems [16] with applications in many areas, such as natural science, engineering technology, and medical care, and it increase the utilization of medical resources and optimize methods of diagnosis or treatment. The MDP theory is also important for medical decision-making, such as the administration of medical devices, admission control in hospitals, decisions on operation timing, and the adjustment of treatment strategies [17-23].

Syndrome differentiation and TCM treatments are very often interdependent and interleaved over time, principally due to uncertainty about the underlying disease, uncertainty associated with patient responses to certain treatments, and the likelihood of patient states varying within the period of treatment, such as from one pattern of TCM to another pattern. The introduction of MDP theory into CER on TCM makes dynamic comparison and evaluation possible. In this study, we show how MDP theory can be used to model integrative medicine treatments (the blending of the best of conventional medicine and complementary and alternative medicine) [24] for patients with acute ischemic stroke (AIS), and to provide an optimal solution from dynamic effectiveness comparisons in sequential clinical practice.

## Methods

### Data collection

The electronic health records (EHR) of patients with AIS hospitalized at the 2^nd ^Affiliated Hospital of Guangzhou University of Chinese Medicine, Guangzhou, China, were collected. The inclusion criteria for the records were a primary diagnosis of cerebral infarction and hospital admission within 14 days of the onset of stroke. Records of patients who had thrombolysis or had undergone early anticoagulation treatment were excluded.

All of the data were collected with an information acquisition form, one form for each record, that captured the general information of the patient, TCM and Western medicine diagnosis, all applied treatments with course detail, levels of neurological function defect on the first, third, and last day of hospitalization, and the results of brain imaging (i.e., computerized X-ray tomography or magnetic resonance imaging). This study was approved by the ethic committee of 2^nd ^Affiliated Hospital of Guangzhou University of Chinese Medicine.

### Description of patients' condition and the criterion to be optimized

To determine the key characteristics for describing the condition of patients with AIS and the criterion to be optimized by using MDP theory, an expert panel was formed that included scholars, physicians of Western medicine, TCM practitioners, and doctors in the field of integrative medicine (with an educational background in both Western medicine and TCM), and a half-day expert panel meeting was held.

Six key characteristics were selected based on the results of the panel meeting (see Additional file 1: Appendix 1): (*i_1_*) age; (*i_2_*) any disease history, such as diabetes, hypertension, coronary heart disease, abnormal blood liquid level, or auricular fibrillation; (*i_3_*) any complication, such as pulmonary infection, urinary tract infection, or deep vein thrombosis; (*i_4_*) TCM diagnosis; (*i_5_*) TCM syndrome differentiation (TCM pattern); and (*i_6_*) level of neurological function (with items for evaluation taken from the NIHSS [25] and assessment standard of neurological function impairment [26]). A score was used to describe the level of neurological function defect (see Additional file 2: Appendix 2). The total scores were in the range of 0-29, where a high score indicates poor function. Patients who were dead scored 29.

Duration of hospitalization for each patient was divided into two stages. Stage 1 ran from admission to the third day of hospital stay, and Stage 2 ran from the third day of hospital stay to discharge. This resulted in three time points for the *state *assessment: the first (*timepoint 1*, *t1*), third (*timepoint 2, t2*), and last day (*timepoint 3, t3*) of hospitalization. Each record was treated as two "s*tate*-a*ction*-*reward*" stages divided by the three timepoints. *State *refers to a patient's condition in terms of the six key characteristics; *action *represents the combination of treatments; and *reward *refers to the value of the differential between the scores for neurological function impairment [25,26] before and after treatment (equal to the total score before treatment minus the score after treatment). According to the expert panel's advice, the total reward values for the two stages became the criteria to be optimized. In terms of the reward values, 0 represents no change in a patient's condition, values larger than 0 represent improvement in a patient's condition, and values lower than 0 mean deterioration. If the value is larger than 0, then the larger the value, the better the improvement in *state*. The *action *that maximizes the total reward value is regarded as the optimal *action*, that is, the optimal intervention combination for the corresponding *state*.

### Description of interventions

Five circumstances were used to distinguish different treatment combinations (*action*) at each stage (see Additional file 3: Appendix 3): (*a_1_*) whether to use antiplatelet and/or anticoagulant agents; (*a_2_*) whether to use TCM treatments for replenishing qi and wen yang (*Yi Qi Wen Yang*); (*a_3_*) whether to use TCM treatments for clearing heat and extinguishing wind (*Qing Re Xi Feng*); (*a_4_*) whether to use TCM treatments for relaxing the bowels; and (*a_5_*) whether to use herbal medicine.

Treatment strategies were carried out at the request of the physician in charge of the patient under the same theory of TCM [27]. Patients with a TCM diagnosis belonging to the *Yin *pattern were treated by "*Yi Qi Wen Yang*" treatments, and those with a TCM diagnosis belonging to the *Yang *pattern received "*Qing Re Xi Feng*" treatments. Herbal medicine was prescribed according to the current symptoms of the patient. If the patient was constipated, TCM treatments to relax the bowels were used. Aspirin or Clopidogrel was taken orally by each patient within 48 hours of hospital admission, except those who were allergic to or genuinely intolerant of these agents. Anticoagulant agents, including unfractionated heparin (UFH), low-molecular-weight heparin (LMWH), or warfarin were used if the patient had any of the following conditions: atrial fibrillation, serious artery angiostenosis, or advancing stroke. Any treatment might be changed at any time if the physician thought it necessary.

For patients with a history of hypertension, diabetes, or dyslipdemia, the agents that they had been taking before admission continued to be administrated during their hospital stay. However, these interventions were not included in the analysis, as they did not focus on stroke treatment.

### Data management and analysis

All of the information acquisition forms were double entered with EpiData 3.1 (EpiData Association Odense, Denmark). The final dataset was converted into SPSS format. Missing data were replaced by the median of nearby points. Data were analyzed primarily with SPSS13.0 (SPSS, USA). The Markov decision processes (MDPs) were written in C language and compiled using Dev C++ 4.9.9.2.

#### Formulating an MDP model for the treatment of AIS

According to clinical experience and TCM theory, treatment decision-making depends on the current condition of patient, and the corresponding TCM/integrative medicine (i.e. the combination of practices and methods of alternative medicine with conventional medicine) therapies are described as non-stationary finite horizon MDPs, in which each *state *variable denotes the patient's condition at a certain time. The optimality problem is solved by maximizing the non-stationary finite horizon expected total utility. For finite horizon MDPs, the *state *space is a set of vectors consisting of all possible conditions for a patient, the set of available *actions *for a *state *is composed of treatments used for therapy for a given *state*, the transition probabilities in the MDPs are determined by the records of therapeutic effectiveness, and the corresponding utility function is evaluated based on the neurological functional impairment score related to the patient's condition and the effectiveness of treatment. Thus, the optimality problem is actually described as a non-stationary finite horizon expected total utility MDP model, and the optimality technique already developed for MDPs can be used to solve it efficiently [16].

#### Formulating a model for MDPs with finite horizon reward criteria

First, it is necessary to specify the condition of the patient, which is the information known by the physician. A *state i *in MDPs denotes the patient's condition. As described in former section, a patient's condition is evaluated based on an overall consideration of various factors, such as *i*_6 _represents level of consciousness, visual field defects, and muscle power of the limbs, etc.. Thus, the *state *is denoted by a vector i = (i_1_, ..., i_n_), where the *state *vector i_k _(k = 1, ..., n) corresponds to every aspect of the patient's condition and *n *is the dimension of the *state *vector. The *state *space is composed of all possible *state *vectors, that is, S = {i = (i_1_, ..., i_n_) | i_k_∈{0, 1, ..., l_i_}, k = 1, ..., n}, where l_i _denotes the number of corresponding factors.

Second, a vector consisting of treatment combinations *a* = (*a*_1_, ..., *a_m_*) is regarded as *action *a available to the decision-maker. As explained in former "*description of intervention*" section, in the treatment of AIS, each component *a_i _*corresponds to a type of treatment used for therapy, and *a_i _*takes a value in {0, 1, ..., j_i_} (i = 1, ..., m). For example, in the case of whether to use antiplatelet agents or not, 0 denotes that an antiplatelet agent should not be used and 1 denotes that aspirin and/or clopidogrel should be chosen. Similarly, in the case of whether to use herbal medicine or not, 0 and 1 respectively denote that herbal medicine should not and should be used. A(i) denotes a set of all possible *actions *available to the controller when the state is at *state i*∈S. In other words, A(i) represents the set of all treatments available to the controller at *state i*.

Third, when a physician prescribes a type of treatment combination (*action a*) for a certain patient in *state i*, the corresponding effectiveness can be detected in *state j *of the patient at the next observable time point. Therapeutic effectiveness may differ when the same treatment combination is applied to different patients with the same condition. Thus, the dynamic evolution of the treatment process is specified using the so-called transition probability P_t_(j|i,a), which means that P_t_(j|i,a) denotes the probability that the *state *is j ∈S at time t + 1 when *action a*∈A(i) is taken at *state i*∈S at time t. We use # (j, i, *a*) to denote the number of transfers from *state i *to the next *state j* under *action a*. For each *state i*, j∈S, and any given *action a*∈A(i), the transition probability is given by Equation (1).

(1) $$p_{t}\left(j|i,a\right):=\frac{\#\left(j,i,a\right)}{\underset{j\in S}{\sum }\#\left(j,i,a\right)}$$

Fourth, the *reward *function u_t_(i, *a*), which depends on the current *state i *∈ S, a chosen *action a*∈A(i), and decision epoch t, is expressed as

(2) $$u_{t}\left(i,a\right)\;=\;\underset{j\in S}{\sum }p_{t}\left(j|i,a\right)u_{t}\left(j,i,a\right),\;$$

where u_t_(j, i,*a*) denotes the *reward *value when the *state *of the treatment process is i at stage t, an *action a*∈A(i) is taken, and the treatment process results in *state j* at the next stage t + 1.

Finally, to complete the model, it is necessary to introduce the N-horizon expected total reward criterion. This needs to define a class of policies (i.e., all possible sequences of treatment combinations) admissible to the controller. A policy can be denoted as a sequence of functions π = {f_1_, f_2_, . . f_N_}, where f_t _(1 ≤ t ≤ N) acts on S and satisfies that f_t_(i)∈A(i) for all i∈S. Hence, function f_t_(i) is the treatment combination chosen at *state i *at stage t. Let Π be the set of all policies. For any given policy π and initial *state i*, J(π,i) denotes the corresponding expected total reward from the initial time to the end time N.

To that end, a model is specified for non-stationary MDPs with the N -horizon expected total reward criterion for the foregoing treatment processes:

(3) $$\left\{S,\left(A\left(i\right),i\;\in \;S\right),\;p_{t}\left(j|i,a\right),u_{t}\left(i,a\right)\right\},\;$$

where the *state *space S, the available *action *set A(i) at *state i*∈S, the transition probability p_t_(j|i,*a*) with i, j∈S and *a*∈A(i), and the *reward *function u_t_(i,*a*), are as previously defined. To elucidate following arguments, some notation is introduced: For each fixed policy π = {f_1_, f_2_, . . f_N_}∈Π, a transition probability matrix P(t, π) is defined with the (i,j) element as p_t_(j|i, f_t_(i)).

For each π∈Π and initial *state i*∈S, the N -horizon expected total reward to be maximized is denoted by

(4) $$J\left(\pi ,i\right):=\;E_{i}^{\pi }\left[\overset{N-1}{\underset{t=0}{\sum }}u_{t}\left(i\left(t\right),a\left(t\right)\right)\;+\;u_{N}\left(i\left(N\right)\right)\right],$$

where $E_{i}^{\pi }$ denotes the expectation operator determined by the given p_t_(j|i, f_t_(i)) and the initial *state i∈*S, *i*(t) and *a*(t) are the *state* and *action* variables at time t, and u_N_(i(N)) is the terminal *reward *associated with the *state i*(N)∈S; see [16] for details.

Finally, the corresponding optimal value function is defined as J*(i) = sup_π∈II_J(π, i), i∈S. A policy π* in Π is said to be optimal if J(π*,i) = J*(i) for all i∈S.

#### Solutions to the optimality problem

For each π∈Π, *U*_t_(π, *i*) denotes the corresponding expected total utility from time t to the end time N given *state i_t _*= *j* at time t, that is (by the well known Markov property),

(5) $$U_{t}\left(\pi ,j\right)\;:=\;E_{i}^{\pi }\left[\overset{N-1}{\underset{n=t}{\sum }}u_{t}\left(i\left(t\right),a\left(t\right)\right)\;+\;u_{N}\left(i\left(N\right))\right|\;i\left(t\right)\;=j\right]\;\text{for}\;t\;=\;N\;-\;1,...,1\;$$

(6) $$U_{N}\left(\pi ,j\right)\;:=\;u_{N}\left(j\right),\;j\;\in S.\;$$

Further,

(7) $$J_{t}\left(i\right):=\underset{\pi \in \text{Π}}{\text{inf}\;}U_{t}\left(\pi ,i\right)\;\text{for}\;t\;=\;N,...,\;1\;$$

implies that J*(i) = *U*_1_(i) = J_1_(i).

To find a method to obtain an optimal policy, by Theorem 4.3.3 (16) the following algorithm is used.

StepI: Set t = N and

(8) $$J_{N}\left(i\right):=u_{N}\left(i\right)\;\text{for}\;\text{all}\;i\;\in \;S\;$$

StepII: Substitute t-1 for t and compute J_t_(i) by

(9) $$J_{t}\left(i\right)\;=\;\underset{a\in A\left(i\right)}{\text{max}}\;\left\{u_{t}\left(i,a\right)\;+\underset{j\in S}{\sum }P_{t}\left(j|i,a\right)J_{t+1}\left(j\right)\right\}\;\text{for}\;t\;=\;N-1,...,1.\;$$

Obtain f_t_*, which realizes the maximum in Eq. (9).

Step III: If t = 1, then stop. Otherwise return to StepII. The policy obtained π* = {f*_1_, ..., f*_N-1_} is optimal (by Theorem 4.3.3 in [16]) as the control model consists of finite *state *and *action *spaces.

#### Numerical implementation

All of the records from the patients with AIS were broadly classified into several groups according to the patient's condition (each of which is called a "*state*"), and the types of treatments were divided into two stages during which different treatment combinations were used. Information was collected to form Tables 1 and 2, which show patient condition and the corresponding treatment combination (i.e., "*actions*") at Stage 1 and Stage 2, respectively. Patient condition as assessed by the six key characteristics is listed in columns 2 through 7. The first column denotes the number of patients with the same condition, and columns 8 through 12 list the main treatments (sometimes more than one for each "*state*") used for AIS (the columns in Tables 1 and 2 have the same meaning but are for a different treatment stage.)

**Table 1:** The patients' conditions and treatments at Stage 1*

No. of cases			*States* at t_1_						*Actions* at t_1_		
	
	*i*_1_	*i*_2_	*i*_3_	*i*_4_	*i*_5_	*i*_6_	*a*_1_	*a*_2_	*a*_3_	*a*_4_	*a*_5_
22	2	0	0	1	1	1	0	0	0	0	1
							0	0	1	0	1
							0	0	1	1	1
							1	0	0	0	1
							1	0	1	0	1
							1	0	1	1	1
16	2	0	0	1	1	2	1	0	0	1	1
							1	0	1	0	1
							1	0	1	1	0
							1	0	1	1	1
							1	1	1	1	1
......	......	......	......	......	......	......	......	......	......	......	......

*stage 1: from timepoint 1 (t_1_) to timepoint 2 (t_2_). (The following the same)

**Table 2:** The patients' conditions and treatments at Stage 2*

**No. of cases**	***States* at t_2_**						***Actions* at t_2_**				
	
	***i*_1_**	***i*_2_**	***i*_3_**	***i*_4_**	***i*_5_**	***i*_6_**	***a*_1_**	***a*_2_**	***a*_3_**	***a*_4_**	***a*_5_**
	
19	2	0	0	1	1	1	0	0	1	0	1
							0	0	1	1	1
							1	0	1	0	1
							1	0	1	1	1
17	2	0	0	1	1	2	1	0	0	0	1
							1	0	0	1	1
							1	0	1	0	1
							1	0	1	1	1
							1	1	1	0	1
......	......	......	......	......	......	......	......	......	......	......	......

*stage 2: from timepoint 2 (t_2_) to timepoint 3 (t_3_). (The following the same)

The elements of the MDP model can now be formulated. From Table 1 and Table 2, the state space can be expressed as S = {200111, 200112, ......, 311122, 311123}, and the corresponding sets of admissible actions are given as

A 200111 = {00001; 00101; 00111; 10001; 10101; 10111}

A (200112 = {10011; 10101; 10110; 10111; 11111; 10001; 11101} ...... The optimality problem is considered to be within a finite time horizon from stage 1 to stage 2. A terminal *reward *of 0 is assigned to all *states*. Based on Tables 1 and 2 and Eq (1), the transition probabilities p_t_(j|i, *a*) (t = 1, 2) are computed and listed in Additional file 4: Appendix 4 and Additional file 5: Appendix 5. From the neurological functional impairment scores in Tables 1 and 2 and Eq (2), the *reward *functions u_t _(i, *a*) (t = 1, 2) can be obtained by Eq (2), and are listed in Additional file 6: Appendix 6 and Additional file 7: Appendix 7.

Using the algorithm to solve the optimal problem, an optimal policy π* = {f*_1_, f_2_*} (corresponding to the optimal treatments) can be obtained as follows.

f *_1 _(200111) = {00001}, f *_1 _(200112) = {10101},......

f *_2 _(200111) = {00111}, f *_2 _(200112) = {10001},...... The optimal treatments with this optimal policy are shown in Table 3 and Table 4.

**Table 3:** Optimal combination of treatment at stage 1 (example)

No. of cases	*States* at t_1_						*Actions* at t_1_				
	***i*_1_**	***i*_2_**	***i*_3_**	***i*_4_**	***i*_5_**	***i*_6_**	***a*_1_**	***a*_2_**	***a*_3_**	***a*_4_**	***a*_5_**

22	2	0	0	1	1	1	0	0	0	0	1
16	2	0	0	1	1	2	1	0	1	0	1
......											

**Table 4:** Optimal combination of treatment at stage 2 (example)

No. of cases	*States* at t_2_									*Actions* at t_2_	
	
	*i*_1_	*i*_2_	*i*_3_	*i*_4_	*i*_5_	*i*_6_	*a*_1_	*a*_2_	*a*_3_	*a*_4_	*a*_5_
19	2	0	0	1	1	1	0	0	1	1	1
17	2	0	0	1	1	2	1	0	0	0	1
......											

## Results

### General information

A total of 1504 records with a primary diagnosis of AIS were identified for the period 1^st ^May 2005 to 31^th ^July 2008. Of these, 1337 met the inclusion criteria. Only *states *with more than 10 (including 10) patients' information were included, resulting in 960 records being enrolled in the MDP model representing 30 kinds of patient condition. Sixty-eight percent of records were from patients over 66 years old. A disease history was given for 74% of the 960 patients. Most of the records had fairly low scores for neurological function impairment, indicating that the severity of the patient's condition was minor to medium (see Table 5). The *i*_6 _value for eight patients who were dead in stage 2 was 29 (the highest score for neurological functional impairment).

**Table 5:** General information of the patients at admission

N = 960	Numbers of cases(n)
**Age**	
46-65 years	308
More than 66 years	652
**Disease history**	
None	252
Have at least one	708
**Complications**	
None	942
Have at least one	18
**TCM Diagnosis**	
Apoplexy involving channels or collaterals	960
**Syndrome differentiation of TCM (Pattern of TCM)**	
*Yang *pettern	305
*Yin *pettern	601
Composite pattern	38
Other patterns	16
**Level of neurological function defect**	
Level 1: 0-2 scores	402
Level 2: 3-5 scores	355
Level 3: 6-12 scores	203
Level 4: 13-19 scores	0
Level 5: 20-29 scores	0

There was 0 to 1.12% of missing data in *i*_1 _to *i*_5 _and 0.07 to 18.39% of data missing for *i*_6_, of which 18.39% was on ataxia, 13.80% information on visual field defects, and 13.76% on sensory disturbance. Other missing data for *i*_6 _were found in other indexes, such as level of consciousness, facial paralysis, muscle power of upper and lower limbs, aphasia, and dysarthria, with levels of missing data ranging from 0.07 to 7.11%. For *a_1 _*to *a_5 _*this figure was 0 to 0.37%. All of the missing data were replaced.

### Optimal combination of treatments for corresponding *states*

By calculating and screening with the MDP theory, the optimal combinations of treatments for the 30 *states *(see Table 6 and Table 7) were obtained.

**Table 6:** Optimal combination of treatments for a variety of states at Stage 1

No. of cases	*States* at t_1_						*Actions* at t_1_					*Rewards* at t_2_*
		
	*i*_1_	*i*_2_	*i*_3_	*i*_4_	*i*_5_	*i*_6_	*a*_1_	*a*_2_	*a*3	*a*_4_	*a*_5_	
130	3	1	0	1	2	2	0	1	0	1	1	4.000
122	3	1	0	1	2	1	0	1	0	1	1	1.000
57	3	1	0	1	1	1	0	0	1	0	1	1.000
51	3	1	0	1	2	3	1	0	0	0	1	6.283
50	2	1	0	1	2	1	1	0	1	0	1	2.000
43	2	1	0	1	2	2	1	1	0	0	1	3.700
41	3	0	0	1	2	1	0	1	0	0	1	0.344
40	3	1	0	1	1	2	0	0	0	0	1	5.000
38	3	1	0	1	1	3	0	0	1	1	1	7.333
35	3	0	0	1	2	2	1	1	0	1	1	1.750
33	3	0	0	1	2	3	1	0	0	0	0	12.000
31	2	1	0	1	1	1	1	0	1	1	1	0.694
30	2	1	0	1	1	2	1	0	1	1	1	2.056
23	2	1	0	1	1	3	0	0	1	1	1	7.000
22	2	0	0	1	1	1	0	0	0	0	1	0.667
22	2	0	0	1	2	1	0	0	0	0	1	1.000
21	2	1	0	1	2	3	1	1	1	1	1	5.571
20	3	0	0	1	1	1	0	0	1	1	1	2.000
18	2	0	0	1	2	2	0	1	0	0	1	1.000
18	3	0	0	1	1	2	0	0	1	1	1	4.500
18	3	1	0	1	3	1	0	1	0	0	1	3.000
17	2	0	0	1	2	3	0	1	0	1	1	4.636
16	2	0	0	1	1	2	1	0	1	0	1	5.200
11	3	1	0	1	3	2	0	1	0	0	1	2.000
10	3	0	0	1	1	3	1	1	1	1	1	6.000
10	3	1	0	1	4	1	0	0	1	1	0	1.500
10	3	1	1	1	2	3	1	1	1	1	1	6.100
9	2	1	0	1	3	1	0	0	1	0	1	2.000
8	3	1	1	1	2	2	0	1	0	0	1	0.000
6	2	1	0	1	4	2	0	0	0	0	1	0.333

*At timepoint 1 (t_1_) ***Actions ***are given to the ***States***, and get ***Rewards ***at t_2_. (The following the same)

**Table 7:** Optimal combination of treatments for a variety of states at Stage 2

Cases	*States* at t_2_						*Actions* at t_2_					*Rewards* at t_3_*
		
	*i*_1_	*i*_2_	*i*_3_	*i*_4_	*i*_5_	*i*_6_	*a*_1_	*a*_2_	*a*_3_	*a*_4_	*a*_5_	
127	3	1	0	1	2	1	0	1	0	1	1	1.000
119	3	1	0	1	2	2	0	0	0	0	1	4.000
60	3	1	0	1	2	3	1	0	0	0	1	4.667
53	2	1	0	1	2	1	1	0	0	0	1	1.000
51	3	1	0	1	1	1	1	0	0	1	1	1.000
42	3	0	0	1	2	1	1	1	0	0	1	0.167
41	3	1	0	1	1	2	1	0	1	1	0	5.000
39	3	1	0	1	1	3	0	1	0	0	1	6.000
38	3	0	0	1	2	3	1	0	0	1	1	9.000
35	2	1	0	1	2	2	1	0	0	0	1	4.000
31	2	1	0	1	1	2	0	0	1	1	1	2.000
30	3	0	0	1	2	2	1	1	0	1	1	1.333
29	2	1	0	1	1	1	1	0	1	0	1	0.500
26	2	1	0	1	2	3	1	1	0	0	1	2.571
23	2	1	0	1	1	3	1	0	0	0	1	7.000
22	2	0	0	1	2	1	1	1	0	0	1	0.667
19	2	0	0	1	1	1	0	0	1	1	1	1.000
19	2	0	0	1	2	2	1	1	0	1	1	2.000
19	3	0	0	1	1	1	0	0	1	0	1	3.000
19	3	0	0	1	1	2	1	1	1	0	1	2.500
18	2	0	0	1	2	3	1	1	0	0	1	1.000
18	3	1	0	1	3	1	1	0	0	0	1	2.636
17	2	0	0	1	1	2	1	0	0	0	1	5.000
12	3	1	1	1	2	3	0	1	0	0	1	3.000
11	3	1	0	1	3	2	1	1	0	0	1	2.000
11	3	1	0	1	4	1	1	0	1	1	1	0.500
9	2	1	0	1	3	1	1	0	0	1	1	2.000
9	3	0	0	1	1	3	1	0	1	1	1	2.000
7	2	1	0	1	4	2	1	0	1	1	1	0.333
6	3	1	1	1	2	2	1	0	1	0	1	0.000

*At timepoint 2 (t_2_) ***Actions ***are given to the ***States***, and get ***Rewards ***at t_3_. (The following the same)

The results of six *states *(see Table 8 and Table 9) can be used as an example to show how these can be used to individually compare the effectiveness of treatments. The *states *in Table 8 represent patients who were older than 66 (*i*_1 _= 3), had at least one kind of disease history (*i*_2 _= 1), were without complications during their hospitalization (*i*_3 _= 0), had *Zhong Jing Luo *(apoplexy involving channels or collaterals) (*i*_4 _= 1) as the TCM diagnosis and a *Yin *TCM pattern (*i*_5 _= 2). Different levels of neurological functional impairment (*i*_6_) were detected, which meant that the severity of stroke varied among patients, as represented by *State *10036, *State *10037, and *State *10038.

**Table 8:** Example of states within which patient's pattern of Chinese medicine was *Yin*

*State*								Stage 1							Stage 2					
							
Code*	*i*_1_	*i*_2_	*i*_3_	*i*_4_	*i*_5_	*i*_6_	Cases	*a*_1_	*a*_2_	*a*_3_	*a*_4_	*a*_5_	*Rewards* at t_2_	Cases	*a*_1_	*a*_2_	*a*_3_	*a*_4_	*a*_5_	*Rewards* at t_3_
(10036)	3	1	0	1	2	1	122	0	1	0	1	1	1.00	127	0	1	0	1	1	1.00
(10037)	3	1	0	1	2	2	130	0	1	0	1	1	4.00	119	0	0	0	0	1	4.00
(10038)	3	1	0	1	2	3	51	1	0	0	0	1	6.28	60	1	0	0	0	1	4.67

*each state is coded difference according to the sequence of composing of the six characteristics.

**Table 9:** Examples of States within which patient's pattern of Chinese medicine was *Yang*

	*State*							Stage 1							Stage 2					
							
Code	*i*_1_	*i*_2_	*i*_3_	*i*_4_	*i*_5_	*i*_6_	Case	*a*_1_	*a*_2_	*a*_3_	*a*_4_	*a*_5_	*Rewards* at t_2_	Case	*a*_1_	*a*_2_	*a*_3_	*a*_4_	*a*_5_	*Rewards* at t_3_
(10031)	3	1	0	1	1	1	57	0	0	1	0	1	1.00	51	1	0	0	1	1	1.00
(10032)	3	1	0	1	1	2	40	0	0	0	0	1	5.00	41	1	0	1	1	0	5.00
(10033)	3	1	0	1	1	3	38	0	0	1	1	1	7.33	39	0	1	0	0	1	6.00

At Stage 1, 122 patients were in *State *10036, and received a combination of therapeutic intervention including TCM treatments to replenish qi and wen yang (*Yi Qi Wen Yang*), TCM treatments to relax the bowels, and herbal medicine (labeled as 01011). Each patient was given a score for neurological functional impairment to describe their *i_6 _*level. Among patients in *State *10036 at Stage 1, those who had been treated with a combination of *a*_2_, *a*_4_, and *a*_5 _(labeled as *action *"01011" at Stage 1) got the highest *Reward *(valued as 1 unit, see Table 8) at t_2 _compared with other kinds of treatment combinations for patients in the same *State*.

One hundred and twenty-seven patients were in *State *10036 at Stage 2, which implies that if the treatment combination labeled "01011" was maintained, then patients in this *State *at Stage 2 would obtain the highest *reward *(1 unit) at t_3_.

Similarly, for patients at Stage 1 in *State *10037, who had a more severe clinical condition than those in *State *10036, the results showed that if the *action *was "01011", then the *reward *value would be a maximum of 4 units. In contrast, for patients in *State *10037 at Stage 2, an intervention with only herbal medicine (*action *labeled as "00001") resulted in the highest *reward *of 4 units. For patients in *State *10038 at Stage 1, a "10001" *action* resulted in a reward of 6.28 units at t_2_, whereas the *action *"10001" at Stage 2 resulted in 4.67 units of *reward *at t_3_.

Patients in *States *10031, 10032, and 10033 (see Table 9) all had a TCM pattern of *Yang*, whereas those in *States *10036, 10037, and 10038 had a TCM pattern of *Yin*.

The results in the first line of Table 9 show that by combining TCM treatments for clearing heat and extinguishing wind (*Qing Re Xi Feng*) (labeled as *a*_3_) with herbal medicine (labeled as *a*_5_), the best reward value at *Stage 1 *for patients in *state *10031 was 1 unit. At Stage 2, patients in the same *state *10031 may have needed a treatment of antiplatelet agents (*a*_1_) together with TCM treatments to relax the bowels (*a*_4_), and *a*_5 _to form the *action *known as "10011" to gain a maximum value reward. It seems that for *State *10033, in which patients tendered to have a more severe clinical condition, the two *actions *that involved TCM therapeutic interventions achieved the best *rewards*.

## Discussion

Based on inpatient EHR, MDPs were applied to describe and analyze the dynamic process of different combinations of TCM treatments and/or integrated treatments of TCM and Western medicine for patients with AIS, and to determine the optimal treatment combination for each *State *by comparing the *rewards *gained from the corresponding *actions*. To the best of our knowledge, no similar topic has been previously addressed in the field of integrative medicine (IM) or in complementary and alternative medicine (CAM).

No medication has yet been confirmed to have neuroprotective effects in the management of patients with AIS [28]. Although antiplatelet agents can reduce the risk of mortality and morbidity when aspirin is administered within 48 hours after the onset of stroke, it cannot be used in up to 28% patients with aspirin "resistance" [29]. The management of patients with AIS with heparin carries an increased risk of bleeding complications [30]. The use of intravenous recombinant tissue plasminogen activators (rt-PA) in cerebral infarctions is associated with improved outcomes, but cannot be used as a routine therapy outside special units [31].

Several commonly used and government-approved traditional Chinese patent medicines (TCPMs), such as, *Ginkgo biloba *[32], milk vetch [33,34], Mailuoning [35], Qingkailing [36], and Danshen [37] agents, have shown promising effects for ischemic stroke. However, no definite conclusions can be drawn from studies of these agents due to a general lack of reporting on methodology [30,38-40]. Properly designed clinical research to study the role of traditional medicine in ischemic stroke is warranted, but a number of issues must be addressed in the design of such studies first [41]. One of these issues is complex interventions involving varying dosages and interactions. Randomized controlled trials (RCTs) are a possible approach to evaluating complex interventions as a whole compared with an appropriate alternative [42], but cannot separate the benefits of different combinations of components. The multi-component structure of treatments is closer to real world practice, especially in therapy for stroke with complex dynamics from onset through progression [43]. Moreover, the model of applying a treatment and conducting it without any change through the whole course of acute stroke is inconsistent with the basic theory of TCM whereby treatment is altered according to syndrome differentiation [15,44].

The results of this study indicate that the new method of MDPs may prove useful for comparative effectiveness research (CER). MDPs can be applied to dynamically compare the effectiveness of various combinations of complex treatments, and may be able to overcome the uncertainties related to individual patients' responses to certain combination of treatments and the uncertainties concerning dynamic changes in treatment for certain patients over the course of disease [21-23,45].

Past research implies that herbal medicine may possess neuroprotective properties [46,47], protect against ischemic reperfusion injury [48,49], reduce edema in the brain [48], improve cerebral microcirculation [33,47], and inhibit apoptosis [50]. Such properties may partly explain the effectiveness of the combinations of treatments identified in this research.

This study has several limitations. First, all of the data were taken from EHR, and missing data are inevitable. The amount of missing data was less than 1.12% in most categories, although 18.39% of missing data was detected in *i*_6_. As *i*_6 _is a key variable in describing the *rewards *of *actions*, the results should be interpreted cautiously because of the possible bias caused by the replacement of missing data. In addition, due to too much variety, different components of herbal medicine were classified as one *action*. As a result, the effectiveness of different prescriptions of herbal medicine is not comparable. Another limitation is that each patient's record was divided into two stages according to three time points, with each episode being regarded as an independent sample when modeled by MDPs. This is consistent with the Markov property of *non-after effect *according to the basic theory of MDPs, but it may, to a certain extent, ignore potential correlations between episodes obtained from the same patient at different stages. Finally, although the key characteristics representing the patient states were based on the results of an expert panel meeting, the states of patients with acute ischemic stroke are variable, and it is likely that some characteristics that might be important for certain patients were missed.

## Conclusion

MDPs can be used as a new method for comparative effectiveness research on TCM. This new approach makes it possible to compare the effectiveness of certain combinations of treatments dynamically by considering *state*, *action*, and *reward *simultaneously. The method can be applied to optimize medical intervention combinations and to support clinical decision-making. However, the optimal interventions obtained by the MDPs in this study require further validation in clinical practice. The results from the MDP model should be interpreted with caution both due to the property of the MDPs themselves and because of possible bias that may have been generated either from the data collection or the data management. Further exploratory studies with MDPs in other areas in which complex interventions involving TCM, Western medicine, or a combination of both are common would be worthwhile.

## Competing interests

The authors declare that they have no competing interests.

## Authors' contributions

DRW, XPG: Study design, analysis and interpretation, drafts and revision of article, and final approval for submission. YFC, JXC, YQZ, MZ: Study design, acquisition of data and clean up the data, revision of article, and final approval for submission. QLL, JHC, YHH, LEY: Study design, analysis the data, drafts and revision of article, and final approval for submission. YBL: Study design, drafts and revision of article, and final approval for submission. All authors read and approved the final manuscript.

## Pre-publication history

The pre-publication history for this paper can be accessed here:

http://www.biomedcentral.com/1471-2288/12/23/prepub

## Supplementary Material

Additional file 1 **Appendix 1**. State, Action and corresponding values. Click here for file

Additional file 2 **Appendix 2**. Clinical Neurological Functional Impairment Assessment for Stroke Patients. Click here for file

Additional file 3 **Appendix 3**. Traditional Chinese Patent Medicine(TCPM) and Western medicine. Click here for file

Additional file 4 **Appendix 4**. transition probability of step 1. Click here for file

Additional file 5 **Appendix 5**. transition probability of step 2. Click here for file

Additional file 6 **Appendix 6**. utility functions of step 1. Click here for file

Additional file 7 **Appendix 7**. utility functions of step 2. Click here for file
