# Supplementary material for: Comparative effectiveness research on patients with acute ischemic stroke using Markov decision processes
Source: BMC Med Res Methodol. 2012 Mar 9;12:23. doi: 10.1186/1471-2288-12-23 (PMC3348070; doi:10.1186/1471-2288-12-23)
Supplement: Additional file 1 — Appendix 1. State, Action and corresponding values. [file 1471-2288-12-23-S1.PDF]

Appendix 1: *State*, *Action* and corresponding values

1. *States* $(i_1, \ldots, i_6)$ and values.

i_1=Age; (1=18-45 years; 2=46-65 years; 3=more than 66 years;)

i_2= Any disease history as diabetes, hypertension, coronary heart disease, abnormal blood liquid level, or auricular fibrillation;(0=None, 1=Have at least one);

i_3= Any complication as pulmonary infection, urinary tract infection or deep vein thrombosis;(0=None, 1=Have at least one);

i_4=TCM diagnosis; (1=apoplexy involving channels or collaterals, 2=apoplexy involving zang-organs fu-organs, 3＝ Other TCM diagnosis;)

i_5= Syndrome differentiation of TCM (Pattern of TCM): (1=*Yang* pattern, 2=*Yin* pattern, 3=Composite pattern; 4=Other pattern;)

i_6= Levels of neurological functional impairment: Level of consciousness (0-3 points), visual field defects (0,2 points), facial paralysis (0,2 points), muscular strength of limb (0-16 points), aphasia (0,2 points), dysarthria (0, 1 points), feels obstacle (0, 1 points), ataxia (0,2 points). The total score is between 0-29 points, with higher score implying severer situation of the patient; Neurological function impairment scores were divided into five grades as level 1(0-2), level 2(3-5), level 3(6-12), level 4 (13-19), level 5 (20-29).

2. *Actions* (i.e. interventions) $(a_1, \ldots, a_5)$ and values

a_1= Used antiplatele or/and anticoagulant agents or not; (0=Unused, 1=Used);

a_2= Used treatments of TCM on replenishing qi and wen yang (*Yi Qi Wen Yang*) or not; (0=Unused, 1=Used one kind of drugs);

a_3= Used treatments of TCM on clearing heat and extinguishing wind (*Qing Re Xi Feng*) or not; (0=Unused, 1=Used one kind of drugs);

a_4= Used treatments of TCM to relax the bowels or not; (0=Unused, 1=Used);

a_5= Used herbal medicine or not. (0=Unused, 1=Used).
