# Supplementary material for: Comparative effectiveness research on patients with acute ischemic stroke using Markov decision processes
Source: BMC Med Res Methodol. 2012 Mar 9;12:23. doi: 10.1186/1471-2288-12-23 (PMC3348070; doi:10.1186/1471-2288-12-23)
Supplement: Additional file 2 — Appendix 2. Clinical Neurological Functional Impairment Assessment for Stroke Patients. [file 1471-2288-12-23-S2.PDF]

Appendix 2: Clinical Neurological Functional Impairment Assessment for Stroke Patients

Clinical Neurological Functional Impairment Assessment for Stroke Patients

Item	Rating Criteria
1.Level of consciousness	0=awake; 1=sleepiness; 2=lethargy; 3=coma;
2.Visual field defects	0=normal; 2=defected
3.Facial paralysis	0=normal; 2=facial paralysis
4.Muscle power of left upper limb	0=level 5; 1=level 4; 2=level 3; 3=level 2; 4= level 1; 4=level 0;
5.Muscle power of left lower limb	0=level 5; 1=level 4; 2=level 3; 3=level 2; 4= level 1; 4=level 0;
6.Muscle power of right upper limb	0=level 5; 1=level 4; 2=level 3; 3=level 2; 4= level 1; 4=level 0;
7.Muscle power of right lower limb	0=level 5; 1=level 4; 2=level 3; 3=level 2; 4= level 1; 4=level 0;
8.Phasia	0=normal; 2= phasia
9.Dysarthria	0=normal; 1= dysarthria
10.Sensory disturbance	0=normal; 1= feels obstacle
11.Ataxia	0=normal; 2= ataxia
**Total scores: 0-29 points**	
