# Supplementary material for: Comparative effectiveness research on patients with acute ischemic stroke using Markov decision processes
Source: BMC Med Res Methodol. 2012 Mar 9;12:23. doi: 10.1186/1471-2288-12-23 (PMC3348070; doi:10.1186/1471-2288-12-23)
Supplement: Additional file 3 — Appendix 3. Traditional Chinese Patent Medicine(TCPM) and Western medicine. [file 1471-2288-12-23-S3.PDF]

Appendix 3: Traditional Chinese Patent Medicine(TCPM) and Western medicine

1. Traditional Chinese Patent Medicine

(1)*YiNaoKang* capsule (*NaoMai* capsule I); *YiNaoKang* oral liquid (*NaoMai* oral liquid I) [§ i];

Each *YiNaoKang* capsule (*NaoMai* capsule I) contained 0.5g of gastrodiae rhizoma, arisaema cum bile, scorpio(天麻、胆南星、全虫),etc. A total of 3 times and 1.5-2g each time were given each day (Goverment-Approved No.: GD Z20071026).

Each *YiNaoKang* oral liquid (*NaoMai* oral liquid I) contained contained 10 ml concentrated liquid of astragali radix, angelicae sinensis, bambusae concretio silicea, scorpi(黄芪、当归尾、天竺黄、全蝎), etc. A total of 3 times and 10-20ml each time were given each day(Goverment-Approved No.:GD Z20071392).

(2)*YiNaoMai* capsule (*NaoMai* capsule II); *YiNaoMai* oral liquid (*NaoMai* oral liquid II) [§ ii];

Each *YiNaoMai* capsule (*NaoMai* capsule II) contained 0.4g of bubali cornu, gentianae radix et rhizoma, bovis calculus artifactus(水牛角、龙胆草、人工牛黄粉), etc. A total of 3 times and 1.2-1.6g each time were given each day(Goverment-Approved No.:GD Z20071033).

Each *YiNaoMai* oral liquid (*NaoMai* oral liquid II) contained contained 10 ml concentrated liquid of pheretima, bambusae concretio silicea, bubali cornu, polyconi cuspidati rhizoma et radix(地龙干、天竺黄、水牛角、虎杖), etc. A total of 3 times and 10-20ml each time were given each day(Goverment-Approved No.:GD Z20071391).

(3)*TongFuXingShen* capsule [§ iii]

Each capsule contained 0.4g of bambusae concretio silicea and bovis calculus artifactu(人工牛黄粉、天竺黄), etc. A total of 3 times and 1.2-1.6g each time were given each day(Goverment-Approved No.:GD Z20071025).

(4)*DengZhanXiXin* injection (Erigeron injection) [i ¶]

The prodcut used were scutellarin ($C_{21}H_{18}O_{12}$, 野黄芩苷) and caffeic acid esters($C_{25}H_{24}O_{12}$, 总咖啡酸酯), extracts of Erigeron, manufactured by Erigeron breviscapus pharmaceutcal Co., LTD., biological valley,YunNan, China.(云南生物谷灯盏花药业有限公司)Each vial 10ml contained 4-6mg scutellari and 20-30mg caffeic acid esters . A total 1-2 times and 20-40ml each time were given each day by Intravenous, slow drip after diluted with 250-500ml of 0.9% sodium chloride injection. (Goverment-Approved No.: Z53021569).

(5)*ShenMai* injection [i]¶

The prodcut used were extracts of ginseng radix et rhizoma rubra 红参 and ophiopogonis radix 麦冬, manufactured by Chiatai Qingchunbao Pharmaceutical Co.,Ltd, Hangzhou, China. A total 1 times and 20-100ml each time were given each day by Intravenous, slow drip after diluted with 250-500ml of 5% glucose injection. (Goverment-Approved No.: Z33020020).

(6)*QingKaiLing* injection [ii]¶

The prodcut used were extracts of cholic acid 胆酸、hyodeoxycholic acid 猪去氧胆酸、bubali cornu 水牛角(粉)、baicalin 黄芩苷、margaritifera concha 珍珠母(粉)、gardeniae fructus 栀子、isatidis radix 板蓝根、 lonicerae japonicae flos 金银花, manufactured by Guangzhou Baiyunshan Mingxing pharmaceutcal Co., LTD., GuangZhou, China.(广州白云山明兴制药有限公司).Each vial 10ml contained 15-32.5mg cholic acid 胆酸 and gardenin 栀子苷>10mg and baicalin 黄芩苷 35-55mg and total nitrogen 总氮 22-30mg. A total 1 times and 20-40ml each time were given each day by Intravenous, slow drip after diluted with 250-500ml of 10% glucose injection or 100ml of 0.9% sodium chloride injection. (Authorized No.: Government-Approved Number Z44022855).

(7)*JinNaDuo* injection [ii]¶

The prodcut used is extract of ginkgo biloba leaves manufactured by Chi Sheng Chemical Co., LTD., Taiwan, China(台湾

济生化学制药厂股份有限公司). Each vial 5ml contained 17.5mg extract of ginkgo biloba leaves and 4.2mg ginkgo flavone glycoside. A total 1-2 times and 10-20ml each time were given each day by Intravenous diluted with 100-200ml of 0.9% sodium chloride injection or 5% glucose injection. (Government approved No.:HC20090014).

§ Approved by the Guangdong Food and Drug Administration for stroke.

¶ Approved by the Chinese State Food and Drug Administration for stroke.

[i] belongs to *Action* 2(a_2).

[ii] belongs to *Action* 3(a_3).

[iii] belongs to *Action* 4(a_4).

2. **Western medicine**

(1)Antiplatelet drug:aspirin / Clopidogrel

(2)Anticoagulant drug: low-Molecular-Weight Heparin(LMWH)/ Warfarin / Unfractionated Heparin (UFH)

(3)Dilatancy blood volume drug: Low Molecular dextran

(4)Neurotrophy drug: Cronassial injection / Cerebrolysin Vial

(5)Cerebral circulation accelerant: alprostadil injection
