# Supplementary material for: Comparative effectiveness research on patients with acute ischemic stroke using Markov decision processes
Source: BMC Med Res Methodol. 2012 Mar 9;12:23. doi: 10.1186/1471-2288-12-23 (PMC3348070; doi:10.1186/1471-2288-12-23)
Supplement: Additional file 5 — Appendix 5. transition probability of step 2. [file 1471-2288-12-23-S5.PDF]

Appendix 5: Transition Probability of Step 2

y<br>(x, a)	200<br>111	200<br>112	200<br>121	200<br>122	200<br>123	210<br>111	210<br>112	210<br>113	210<br>121	210<br>122	210<br>123	210<br>131	210<br>142	300<br>111	300<br>112	300<br>113	300<br>121	300<br>122	300<br>123	310<br>111	310<br>112	310<br>113	310<br>121	310<br>122	310<br>123	310<br>131	310<br>132	310<br>141	311<br>122	311<br>123
(200111, 00101)	1																													
(200111, 00111)	1																													
(200111, 10101)	1																													
(200111, 10111)	1																													
(200112, 10001)	1																													
(200112, 10011)		1																												
(200112, 10101)	0.714	0.286																												
(200112, 10111)	0.286	0.714																												
(200112, 11101)	1																													
(200121, 01001)			1																											
(200121, 01011)			1																											
(200121, 10001)	0.5		0.5																											
(200121, 10011)			1																											
(200121, 11001)			1																											
(200122, 01001)				1																										
(200122, 10011)				1																										
(200122, 11001)			0.462	0.538																										
(200122, 11011)			1.0																											
(200123, 01001)				1																										
(200123, 01011)				1																										
(200123, 11001)			0.182	0.273	0.545																									
(200123, 11011)					1																									
(210111, 00111)						0.5			0.5																					

y	200	200	200	200	200	210	210	210	210	210	210	210	210	300	300	300	300	300	300	310	310	310	310	310	310	310	310	310	311	311
(x, a)	111	112	121	122	123	111	112	113	121	122	123	131	142	111	112	113	121	122	123	111	112	113	121	122	123	131	132	141	122	123
(210111, 10101)						0.875	0.125																							
(210111, 10111)						1																								
(210111, 11111)						1																								
(200112, 00101)							0.5		0.5																					
(200112, 00111)						1																								
(200112, 10011)							1																							
(200112, 10101)						0.6	0.4																							
(200112, 10111)						0.333	0.571	0.048		0.048																				
(200112, 11111)						1																								
(210113, 00101）								1																						
(210113, 00111)								1																						
(210113, 10001)							1																							
(210113, 10011）								1																						
(210113, 10101)						0.333	0.667																							
(210113, 10111)							0.214	0.786																						
(210113, 11101)							1																							
(210121, 00111)										1																				
(210121, 01001)									0.8		0.2																			
(210121, 01011)									1																					
(210121, 10001)									1																					
(210121, 10011)									1																					
(210121, 11001)									0.941	0.059																				
(210121, 11011)									1																					

y (x, a)	200 111	200 112	200 121	200 122	200 123	210 111	210 112	210 113	210 121	210 122	210 123	210 131	210 142	300 111	300 112	300 113	300 121	300 122	300 123	310 111	310 112	310 113	310 121	310 122	310 123	310 131	310 132	310 141	311 122	311 123
(210121, 11101)									1																					
(210121, 11111)									1																					
(210122, 01001)									0.333	0.667																				
(210122, 10001)									1																					
(210122, 10011)										1																				
(210122, 10111)									0.333	0.667																				
(210122, 11000)									1																					
(210122, 11001)						0.042			0.375	0.583																				
(210122, 11011)									1																					
(210122, 11101)									1																					
(210123, 00000)											1																			
(210123, 01001)										0.333	0.667																			
(210123, 10001)									0.333		0.667																			
(210123, 11000)											1																			
(210123, 11001)										0.643	0.357																			
(210123, 11011)										0.75	0.25																			
(210131, 00101)						1																								
(210131, 01001)												1																		
(210131, 10011)												1																		
(210131, 11001)												1																		
(210142, 00001)													1																	
(210142, 10001)													1																	

y<br>(x, a)	200<br>111	200<br>112	200<br>121	200<br>122	200<br>123	210<br>111	210<br>112	210<br>113	210<br>121	210<br>122	210<br>123	210<br>131	210<br>142	300<br>111	300<br>112	300<br>113	300<br>121	300<br>122	300<br>123	310<br>111	310<br>112	310<br>113	310<br>121	310<br>122	310<br>123	310<br>131	310<br>132	310<br>141	311<br>122	311<br>123
(210142, 10111)													1																	
(210142, 11001)													1																	
(210142, 11011)													1																	
(300111, 00101)														1																
(300111, 00111)														1																
(300111, 10001)														1																
(300111, 10011)														1																
(300111, 10101)														1																
(300111, 10111)														1																
(300111, 11101)														1																
(300112, 00101)															0.5	0.5														
(300112, 00111)														0.5	0.5															
(300112, 10011)														1.0																
(300112, 10101)															1															
(300112, 10111)														0.4	0.5	0.1														
(300112, 11101)														1																
(300112, 11111)															1															
(300113, 00111)																1														
(300113, 10101)																1														
(300113, 10111)															0.333	0.667														
(300121, 00011)																	1													
(300121, 01001)														0.143			0.857													
(300121, 10001)																	1													

y / (x, a)	200 111	200 112	200 121	200 122	200 123	210 111	210 112	210 113	210 121	210 122	210 123	210 131	210 142	300 111	300 112	300 113	300 121	300 122	300 123	3101 11	3101 12	310 113	310 121	310 122	310 123	310 131	310 132	310 141	311 122	311 123
(300121, 11001)																	0.967	0.033												
(300121, 11011)																	1													
(300121, 11111)																	1													
(300122, 01001)																	0.25	0.75												
(300122, 10001)																		0.5	0.5											
(300122, 11001)																	0.381	0.571	0.048											
(300122, 11011)																	0.667		0.333											
(300123, 00000)																			1											
(300123, 00001)																			1											
(300123, 01001)																	0.167	0.333	0.5											
(300123, 01011)																		1												
(300123, 10000)																		1												
(300123, 10001)																	0.2	0.4	0.4											
(300123, 10011)																	1													
(300123, 10101)																			1											
(300123, 11001)																	0.188	0.563	0.25											
(300123, 11011)																	0.2		0.8											
(310111, 00011)																				1										
(310111, 00101)																				1										
(310111, 00111)																				0.667	0.333									
(310111, 01001)																				1										
(310111, 01111)																					1									
(310111, 10011)																				1										

y (x, a)	200 111	200 112	200 121	200 122	200 123	210 111	210 112	210 113	210 121	210 122	210 123	210 131	210 142	300 111	300 112	300 113	300 121	300 122	300 123	3101 11	3101 12	3101 13	3101 21	3101 22	310 123	310 131	310 132	310 141	311 122	311 123
(310111, 10101)																				0.857	0.071		0.071							
(310111, 10111)																				0.96	0.04									
(310112, 00001)																					1									
(310112, 00101)																					0.5	0.5								
(310112, 00111)																				0.75	0.25									
(310112, 10001)																					1									
(310112, 10101)																				0.714	0.286									
(310112, 10110)																				1										
(310112, 10111)																				0.583	0.375	0.042								
(310112, 11111)																				1										
(310113, 00101)																					0.333	0.667								
(310113, 00111)																				0.333	0.333	0.333								
(310113, 01001)																				1										
(310113, 10001)																						1								
(310113, 10101)																				0.125	0.5	0.375								
(310113, 10110)																					1									
(310113, 10111)																				0.091	0.364	0.545								
(310121, 01001)																							1							
(310121, 01011)																							1							
(310121, 10001)																							1							
(310121, 10011)																							1							
(310121, 10101)																							1							
(310121, 11001)																							0.978	0.022						

y<br>(x, a)	200<br>111	200<br>112	200<br>121	200<br>122	200<br>123	210<br>111	210<br>112	210<br>113	210<br>121	210<br>122	210<br>123	210<br>131	210<br>142	300<br>111	300<br>112	300<br>113	300<br>121	300<br>122	300<br>123	3101<br>11	310<br>112	310<br>113	3101<br>21	3101<br>22	3101<br>23	310<br>131	3101<br>32	310<br>141	311<br>122	311<br>123
(310121, 11011)																							0.857	0.071			0.071			
(310122, 00001)																							1							
(310122, 01001)																							0.533	0.333	0.133					
(310122, 01011)																							0.333	0.667						
(310122, 10000)																								1						
(310122, 10001)																							0.5	0.5						
(310122, 10011)																							1							
(310122, 10111)																				0.333				0.667						
(310122, 11000)																								1						
(310122, 11001)																							0.522	0.463	0.015					
(310122, 11011)																							0.357	0.643						
(310122, 11101)																								1						
(310123, 01001)																							0.4	0.4	0.2					
(310123, 01011)																									1					
(310123, 10001)																							0.333	0.667						
(310123, 10011)																									1					
(310123, 10111)																								1						
(310123, 11001)																				0.025			0.125	0.25	0.6					
(310123, 11011)																							0.333	0.333	0.333					
(310123, 11101)																									1					
(310131, 01001)																										1				
(310131, 10001)																										1				
(310131, 10101)																										1				

y<br>(x, a)	200<br>111	200<br>112	200<br>121	200<br>122	200<br>123	210<br>111	210<br>112	210<br>113	210<br>121	210<br>122	210<br>123	210<br>131	210<br>142	300<br>111	300<br>112	300<br>113	300<br>121	300<br>122	300<br>123	310<br>111	310<br>112	310<br>113	310<br>121	310<br>122	310<br>123	3101<br>31	3101<br>32	3101<br>41	3111<br>22	3111<br>23
(310131, 11001)																										1				
(310131, 11011)																										1				
(310131, 11101)																										1				
(310132, 01001)																										1				
(310132, 10001)																										0.5	0.5			
(310132, 11001)																										0.6	0.4			
(310132, 11011)																											1			
(310141, 00111)																												1		
(310141, 01101)																												1		
(310141, 10101)																												1		
(310141, 10110)																												1		
(310141, 10111)																												1		
(310141, 11001)																										0.25		0.75		
(311122, 10101)																													1	
(311122, 11001)																													0.75	0.25
(311122, 11011)																													1	
(311123, 00101)																														1
(311123, 01001)																													1	
(311123, 11001)																													0.2	0.8
(311123, 11011)																													0.2	0.8
