# Supplementary material for: Comparative effectiveness research on patients with acute ischemic stroke using Markov decision processes
Source: BMC Med Res Methodol. 2012 Mar 9;12:23. doi: 10.1186/1471-2288-12-23 (PMC3348070; doi:10.1186/1471-2288-12-23)
Supplement: Additional file 6 — Appendix 6. utility functions of step 1. [file 1471-2288-12-23-S6.PDF]

Appendix 6: Utility Functions of Step 1

$u_1(x,a)$		u_1 (x,a)	
u_1 (200111, 00001)	0	u_1 (210121, 00111)	0.500
u_1 (200111, 00101)	-1	u_1 (210121, 01001)	0.000
u_1 (200111, 00111)	-1	u_1 (210121, 01011)	0.000
u_1 (200111, 10001)	0	u_1 (210121, 01101)	0.000
u_1 (200111, 10101)	-0.286	u_1 (210121, 10001)	0.000
u_1 (200111, 10111)	-0.111	u_1 (210121, 10011)	-2.000
u_1 (200112, 10011)	-2	u_1 (210121, 10101)	-2.000
u_1 (200112, 10011)	0.2	u_1 (210121, 11001)	-0.226
u_1 (200112, 10101)	0.000	u_1 (210121, 11011)	0.400
u_1 (200112, 10110)	-0.125	u_1 (210122, 01001)	-0.167
u_1 (200112, 10111)	0.000	u_1 (210122, 10001)	-1.000
u_1 (200121, 00000)	-1.000	u_1 (210122, 10111)	0.000
u_1 (200121, 00001)	0.000	u_1 (210122, 11001)	0.133
u_1 (200121, 01001)	-0.250	u_1 (210122, 11011)	-0.333
u_1 (200121, 10011)	0.000	u_1 (210123, 00000)	0.000
u_1 (200121, 10101)	0.000	u_1 (210123, 01001)	0.000
u_1 (200121, 11001)	-1.091	u_1 (210123, 10001)	0.000
u_1 (200121, 11011)	-1.000	u_1 (210123, 11000)	1.000
u_1 (200121, 11101)	0.000	u_1 (210123, 11001)	-0.500
u_1 (200122, 01001)	0.500	u_1 (210123, 11011)	0.000
u_1 (200122, 10011)	0.000	u_1 (210123, 11111)	3.000
u_1 (200122, 11001)	0.308	u_1 (210131, 00101)	0.000
u_1 (200123, 01001)	0.000	u_1 (210131, 01001)	0.000
u_1 (200123, 01011)	2.000	u_1 (210131, 10011)	-2.000
u_1 (200123, 11001)	0.182	u_1 (210131, 11001)	0.000
u_1 (200123, 11011)	0.000	u_1 (210142, 00001)	0.000
u_1 (200123, 11101)	0.000	u_1 (210142, 10001)	0.000
u_1 (210111, 00111)	0.000	u_1 (210142, 10111)	-0.333
u_1 (210111, 01001)	0.000	u_1 (210142, 11000)	0.000
u_1 (210111, 10101)	-0.700	u_1 (300111, 00111)	0.000
u_1 (210111, 10111)	-0.056	u_1 (300111, 10001)	0.000
u_1 (210111, 11111)	0.000	u_1 (300111, 10101)	0.500
u_1 (210112, 00101)	0.000	u_1 (300111, 10111)	-0.364
u_1 (210112, 00111)	0.000	u_1 (300111, 11101)	0.000
u_1 (210112, 10101)	-1.000	u_1 (300112, 00101)	0.000
u_1 (210112, 10111)	0.500	u_1 (300112, 00111)	2.000
u_1 (210112, 11111)	0.333	u_1 (300112, 10101)	0.000
u_1 (210113, 00111)	0.000	u_1 (300112, 10111)	-0.455
u_1 (210113, 01101)	0.000	u_1 (300112, 11101)	-2.000
u_1 (210113, 10101)	1.333	u_1 (300113, 00101)	3.000
u_1 (210113, 10111)	0.438	u_1 (300113, 00111)	0.000
u_1 (210113, 11011)	0.000	u_1 (300113, 10101)	0.000

u_1 (x,a)		u_1 (x,a)	
u_1 (300113, 10111)	0.400	u_1 (310113, 10111)	0.130
u_1 (300113, 11111)	4.000	u_1 (310113, 11011)	0.000
u_1 (300121, 00011)	0.000	u_1 (310121, 00001)	0.000
u_1 (300121, 01001)	0.111	u_1 (310121, 01001)	-0.150
u_1 (300121, 10001)	0.000	u_1 (310121, 01011)	0.000
u_1 (300121, 10111)	-1.000	u_1 (310121, 10001)	-0.333
u_1 (300121, 11001)	-0.536	u_1 (310121, 10101)	0.000
u_1 (300121, 11011)	-2.000	u_1 (310121, 10111)	-2.000
u_1 (300122, 01001)	-0.500	u_1 (310121, 11001)	-0.134
u_1 (300122, 10001)	0.000	u_1 (310121, 11011)	-0.400
u_1 (300122, 11001)	-0.217	u_1 (310121, 11101)	-1.000
u_1 (300122, 11011)	0.750	u_1 (310122, 01001)	0.136
u_1 (300123, 00000)	0.000	u_1 (310122, 01011)	0.000
u_1 (300123, 00001)	-1.000	u_1 (310122, 10000)	0.000
u_1 (300123, 01001)	0.667	u_1 (310122, 10001)	-0.556
u_1 (300123, 01011)	-2.000	u_1 (310122, 10010)	0.000
u_1 (300123, 10000)	3.000	u_1 (310122, 10011)	0.000
u_1 (300123, 10001)	1.333	u_1 (310122, 10101)	0.000
u_1 (300123, 11001)	0.850	u_1 (310122, 10111)	-0.500
u_1 (300123, 11011)	-0.667	u_1 (310122, 11001)	-0.269
u_1 (310111, 00101)	0.000	u_1 (310122, 11011)	0.800
u_1 (310111, 00111)	-2.286	u_1 (310122, 11101)	0.000
u_1 (310111, 01011)	0.000	u_1 (310122, 11111)	0.000
u_1 (310111, 10001)	0.000	u_1 (310123, 00001)	0.000
u_1 (310111, 10011)	0.000	u_1 (310123, 01001)	0.750
u_1 (310111, 10101)	-0.600	u_1 (310123, 01011)	0.000
u_1 (310111, 10111)	-0.310	u_1 (310123, 10001)	5.000
u_1 (310111, 11001)	0.000	u_1 (310123, 10011)	0.000
u_1 (310112, 00001)	0.000	u_1 (310123, 11001)	0.625
u_1 (310112, 00101)	-1.000	u_1 (310123, 11011)	0.000
u_1 (310112, 00111)	0.000	u_1 (310131, 01001)	0.000
u_1 (310112, 10001)	-1.000	u_1 (310131, 10001)	-1.500
u_1 (310112, 10101)	0.375	u_1 (310131, 11001)	0.000
u_1 (310112, 10110)	2.000	u_1 (310131, 11011)	0.000
u_1 (310112, 10111)	-0.900	u_1 (310132, 01001)	0.000
u_1 (310112, 11011)	0.000	u_1 (310132, 10000)	0.000
u_1 (310112, 11111)	0.000	u_1 (310132, 10001)	0.000
u_1 (310113, 00101)	0.333	u_1 (310132, 11001)	0.000
u_1 (310113, 00111)	1.333	u_1 (310132, 11011)	0.000
u_1 (310113, 10001)	0.000	u_1 (310141, 00110)	1.000
u_1 (310113, 10101)	0.000	u_1 (310141, 00111)	0.000
u_1 (310113, 10110)	2.000	u_1 (310141, 01101)	0.000

u_1 (x,a)		u_1 (x,a)	
u_1 (310141, 10101)	0.000		
u_1 (310141, 10111)	0.000		
u_1 (310141, 11001)	-0.250		
u_1 (311122, 01001)	0.000		
u_1 (311122, 10101)	0.000		
u_1 (311122, 11001)	0.000		
u_1 (311122, 11011)	-4.000		
u_1 (311123, 00101)	0.000		
u_1 (311123, 01001)	0.333		
u_1 (311123, 11001)	0.600		
u_1 (311123, 11111)	3.000		
