# Supplementary material for: Comparative effectiveness research on patients with acute ischemic stroke using Markov decision processes
Source: BMC Med Res Methodol. 2012 Mar 9;12:23. doi: 10.1186/1471-2288-12-23 (PMC3348070; doi:10.1186/1471-2288-12-23)
Supplement: Additional file 7 — Appendix 7. utility functions of step 2. [file 1471-2288-12-23-S7.PDF]

Appendix 7: Utility Functions of Step 2

$u_2(x,a)$		$u_2(x,a)$	
u_2 (200111, 00101)	0.000	u_2 (210121, 10001)	1.000
u_2 (200111, 00111)	1.000	u_2 (210121, 10011)	0.000
u_2 (200111, 10101)	0.333	u_2 (210121, 11001)	0.147
u_2 (200111, 10111)	0.636	u_2 (210121, 11011)	0.400
u_2 (200112, 10001)	5.000	u_2 (210121, 11101)	0.000
u_2 (200112, 10011)	2.000	u_2 (210121, 11111)	0.000
u_2 (200112, 10101)	1.000	u_2 (210122, 01001)	0.667
u_2 (200112, 10111)	1.000	u_2 (210122, 10001)	4.000
u_2 (200112, 11101)	4.000	u_2 (210122, 10011)	0.000
u_2 (200121, 01001)	0.333	u_2 (210122, 10111)	2.000
u_2 (200121, 01011)	0.000	u_2 (210122, 11000)	1.000
u_2 (200121, 10001)	0.000	u_2 (210122, 11001)	0.792
u_2 (200121, 10011)	0.000	u_2 (210122, 11011)	1.000
u_2 (200121, 11001)	0.667	u_2 (210122, 11101)	3.000
u_2 (200122, 01001)	0.000	u_2 (210123, 00000)	1.000
u_2 (200122, 10011)	0.000	u_2 (210123, 01001)	1.333
u_2 (200122, 11001)	1.385	u_2 (210123, 10001)	2.000
u_2 (200122, 11011)	2.500	u_2 (210123, 11000)	0.000
u_2 (200123, 01001)	1.000	u_2 (210123, 11001)	2.571
u_2 (200123, 01011)	1.000	u_2 (210123, 11011)	3.000
u_2 (200123, 11001)	2.636	u_2 (210131, 00101)	-2.000
u_2 (200123, 11011)	1.200	u_2 (210131, 01001)	0.000
u_2 (210111, 00111)	-1.000	u_2 (210131, 10011)	2.000
u_2 (210111, 10101)	0.500	u_2 (210131, 11001)	0.167
u_2 (210111, 10111)	0.353	u_2 (210142, 00001)	0.000
u_2 (210111, 11111)	-1.000	u_2 (210142, 10001)	0.000
u_2 (200112, 00101)	1.500	u_2 (210142, 10111)	0.333
u_2 (200112, 00111)	2.000	u_2 (210142, 11001)	0.000
u_2 (200112, 10011)	-1.000	u_2 (210142, 11011)	0.000
u_2 (200112, 10101)	2.000	u_2 (300111, 00101)	2.000
u_2 (200112, 10111)	0.857	u_2 (300111, 00111)	0.000
u_2 (200112, 11111)	2.000	u_2 (300111, 10001)	0.000
u_2 (210113, 00101)	1.000	u_2 (300111, 10011)	0.000
u_2 (210113, 00111)	0.000	u_2 (300111, 10101)	0.333
u_2 (210113, 10001)	7.000	u_2 (300111, 10111)	0.300
u_2 (210113, 10011)	3.000	u_2 (300111, 11101)	2.000
u_2 (210113, 10101)	4.667	u_2 (300112, 00101)	-1.500
u_2 (210113, 10111)	1.643	u_2 (300112, 00111)	0.500
u_2 (210113, 11101)	2.500	u_2 (300112, 10011)	1.000
u_2 (210121, 00111)	-1.000	u_2 (300112, 10101)	0.000
u_2 (210121, 01001)	-1.000	u_2 (300112, 10111)	2.100
u_2 (210121, 01011)	0.500	u_2 (300112, 11101)	3.000

u_2 (x,a)		u_2 (x,a)	
u_2 (300112, 11111)	0.000	u_2 (310113, 01001)	6.000
u_2 (300113, 00111)	-2.000	u_2 (310113, 10001)	-29.000
u_2 (300113, 10101)	1.500	u_2 (310113, 10101)	3.250
u_2 (300113, 10111)	2.000	u_2 (310113, 10110)	-29.000
u_2 (300121, 00011)	-1.000	u_2 (310113, 10111)	1.955
u_2 (300121, 01001)	0.143	u_2 (310121, 01001)	0.813
u_2 (300121, 10001)	0.000	u_2 (310121, 01011)	1.000
u_2 (300121, 11001)	0.167	u_2 (310121, 10001)	0.333
u_2 (300121, 11011)	0.000	u_2 (310121, 10011)	0.000
u_2 (300121, 11111)	0.000	u_2 (310121, 10101)	0.000
u_2 (300122, 01001)	0.750	u_2 (310121, 11001)	0.422
u_2 (300122, 10001)	-1.000	u_2 (310121, 11011)	-0.071
u_2 (300122, 11001)	0.762	u_2 (310122, 00001)	4.000
u_2 (300122, 11011)	1.333	u_2 (310122, 01001)	0.933
u_2 (300123, 00000)	0.000	u_2 (310122, 01011)	1.000
u_2 (300123, 00001)	-2.000	u_2 (310122, 10000)	0.000
u_2 (300123, 01001)	2.000	u_2 (310122, 10001)	1.600
u_2 (300123, 01011)	4.000	u_2 (310122, 10011)	2.000
u_2 (300123, 10000)	2.000	u_2 (310122, 10111)	1.667
u_2 (300123, 10001)	2.800	u_2 (310122, 11000)	-29.000
u_2 (300123, 10011)	9.000	u_2 (310122, 11001)	1.493
u_2 (300123, 10101)	1.000	u_2 (310122, 11011)	1.143
u_2 (300123, 11001)	3.063	u_2 (310122, 11101)	0.000
u_2 (300123, 11011)	3.400	u_2 (310123, 01001)	3.000
u_2 (310111, 00011)	0.000	u_2 (310123, 01011)	0.667
u_2 (310111, 00101)	0.667	u_2 (310123, 10001)	4.667
u_2 (310111, 00111)	-0.667	u_2 (310123, 10011)	1.000
u_2 (310111, 01001)	0.000	u_2 (310123, 10111)	2.000
u_2 (310111, 01111)	-29.000	u_2 (310123, 11001)	1.950
u_2 (310111, 10011)	1.000	u_2 (310123, 11011)	2.333
u_2 (310111, 10101)	0.071	u_2 (310123, 11101)	0.000
u_2 (310111, 10111)	0.280	u_2 (310131, 01001)	0.667
u_2 (310112, 00001)	0.000	u_2 (310131, 10001)	1.000
u_2 (310112, 00101)	-1.500	u_2 (310131, 10101)	0.000
u_2 (310112, 00111)	1.750	u_2 (310131, 11001)	0.364
u_2 (310112, 10001)	-29.000	u_2 (310131, 11011)	0.000
u_2 (310112, 10101)	1.143	u_2 (310131, 11101)	0.000
u_2 (310112, 10110)	5.000	u_2 (310132, 01001)	1.000
u_2 (310112, 10111)	1.500	u_2 (310132, 10001)	1.500
u_2 (310112, 11111)	3.000	u_2 (310132, 11001)	2.000
u_2 (310113, 00101)	1.000	u_2 (310132, 11011)	0.000
u_2 (310113, 00111)	-5.333	u_2 (310141, 00111)	-0.500

u_2 (x,a)		u_2 (x,a)	
u_2 (310141, 01101)	0.000		
u_2 (310141, 10101)	0.000		
u_2 (310141, 10110)	0.000		
u_2 (310141, 10111)	0.500		
u_2 (310141, 11001)	0.250		
u_2 (311122, 10101)	0.000		
u_2 (311122, 11001)	-1.250		
u_2 (311122, 11011)	0.000		
u_2 (311123, 00101)	-1.000		
u_2 (311123, 01001)	3.000		
u_2 (311123, 11001)	1.600		
u_2 (311123, 11011)	1.800		
